# A systematic review and meta-analysis of vertical transmission route of HIV in Ethiopia

**DOI:** 10.1186/s12879-018-3189-3

**Published:** 2018-06-22

**Authors:** Aklilu Endalamaw, Amare Demsie, Setegn Eshetie, Tesfa Dejenie Habtewold

**Affiliations:** 1 Department of Pediatrics and Child Health Nursing, School of Nursing, College of Medicine and Health Sciences, University of Gondar, P.O.BOX: 196 Gondar, Ethiopia; 2 Department of Medical Microbiology, School of Biomedical and Laboratory Sciences, College of Medicine and Health Sciences, University of Gondar, Gondar, Ethiopia; 3 University of Groningen, University Medical Center Groningen, University Center for Psychiatry, Rob Giel Research Centre, Groningen, The Netherlands; 4 University Medical Center Groningen, Department of Epidemiology, University of Groningen, Groningen, The Netherlands

**Keywords:** HIV-exposed infants, HIV transmission, Infants, Meta-analysis, Mothers, Ethiopia

## Abstract

**Background:**

The burden of mother-to-child transmission rate of HIV is high and risk factors are common in Ethiopia. This systematic review and meta-analysis intended to provide the pooled estimation of mother-to-child transmission rate and its risk factors in Ethiopia.

**Methods:**

We searched PubMed, Google Scholar, EMBASE and Web of Science electronic databases for all available references. We included observational studies including case-control, cohort, and cross-sectional studies. The search was further limited to studies conducted in Ethiopia and publish in English. Heterogeneity was checked using the I^2^ statistic. Egger’s test and the funnel plot were used to assess publication bias. A meta-analysis using a weighted inverse variance random-effects model was performed.

**Results:**

A total of 18 studies with 6253 individuals were included in this systematic review and meta-analysis. Of these, 14 studies with 4624 individuals were used to estimate the prevalence. The estimated pooled prevalence of mother-to-child transmission of HIV was 11.4% (95% CI = 9.1–13.7). The pooled adjusted odds ratio (AOR) of mother-to-child transmission of HIV for the infants from rural area was 3.8 (95% CI = 1.4 to 6.3), infants delivered at home was 3.2 (95% CI = 1.2 to 5.2), infant didn’t take antiretroviral prophylaxis was 5.8 (95% CI = 1.5 to 10.3), mother didn’t take antiretroviral prophylaxis was 6.1 (95% CI = 2.5 to 9.6), mothers didn’t receive PMTCT intervention was 5.1 (95% CI = 1.6, 8.6), and on mixed feeding was 4.3 (95% CI = 1.8 to 6.7).

**Conclusions:**

This systematic review and meta-analysis showed that mother-to-child transmission rate of HIV was high in Ethiopia. Being from the rural residence, home delivery, not taking antiretroviral prophylaxis, the absence of PMTCT intervention, and mixed infant feeding practices increased the risk of HIV transmission.

**Trial registration:**

It is registered in the Prospero database: (PROSPERO 2017: CRD42017078232).

**Electronic supplementary material:**

The online version of this article (10.1186/s12879-018-3189-3) contains supplementary material, which is available to authorized users.

## Background

The pandemics of Human Immunodeficiency Virus (HIV) have been affecting many segments of the population all over the world [1]. In 2016, around 36.7 million people lived with HIV [2]. In sub-Saharan Africa and globally, 110,000 and 160,000 children got new HIV infection respectively [2, 3].

HIV has many routes of transmission including mother-to-child transmission (MTCT) [4]. More than 90% of children acquired HIV through MTCT [5, 6], predominantly high in Africa [7]. Studies reported that MTCT rate during pregnancy or postpartum period was 23% [8] even though it varies from 15 to 45% in the absence of prophylaxis [9]. Between 2009 and 2013, the MTCT rate reduced from 28 to 18% in sub-Saharan Africa [10].

To control MTCT of HIV as part of end Acquired Immunodeficiency Syndrome epidemic strategy [11, 12], several activities have been inaugurated in Ethiopia. Ethiopia implemented prevention of mother to child transmission (PMTCT) intervention since 2001 [13], such as increasing institutional delivery, Antiretroviral coverage, infant prophylaxis, and proper feeding practices of infants [14, 15].

However, in 2016, 40, 11, and 26% of Ethiopian women ever screened for HIV, started mixed feeding for infants before 6 months and delivered at health institutions, respectively [16]. Consequently, MTCT of HIV occurred significantly. Similarly, there were an estimated 14,000 HIV-positive newborns in Ethiopia [17].

Despite many efforts to study the prevalence and risk factors of MTCT of HIV, there are still fragmented primary studies in Ethiopia [18–35]. The majority of the studies showed epidemiologic variations from 0.7% [28] to 32.0% [19] over time and across geographical areas. Similarly, a disagreement among those studies about major factors was observed. Therefore, this systematic review and meta-analysis aimed to provide a pooled national estimate of the prevalence of MTCT of HIV and its associated factors in Ethiopia. The result of this study may help to guide policy and decision makers in the prevention and control of MTCT of HIV.

## Methods

### Reporting

The Preferred Reporting Items for Systematic Reviews and Meta-analyses (PRISMA) guideline [36] was used to report the result of this systematic review and meta-analyses (Additional file 1).

### Databases and searching strategy

We searched PubMed, Web of Science, Excerpta Medica Database (EMBASE), Google Scholar and psycEXTRA databases for all available studies using the following search terms: “HIV”; “human immunodeficiency virus”; “AIDS”; “Acquired immunodeficiency syndrome”; “mother to child”; “mother to infant”; “mother”; “infant”; “newborn”; “neonate”; “baby”; “child”; “vertical transmission”; “mother-to-child transmission”; “MTCT”; “transmission”; “PMTCT”; “prevention of mother to child transmission”; “factors”; “determinants”; “predictors”; “enablers”; “barriers”; and “Ethiopia”. Search string was developed using “AND” and “OR” Boolean operators. An example of the search details for PubMed illustrated in Additional file 2. Grey literatures were also searched from Ethiopian’s University (University of Gondar and Addis Ababa University) research repository online library. In addition, a manual search of the reference lists of included articles was performed.

### Inclusion and exclusion criteria

The studies were included if they met the following inclusion criteria: (1) observational studies, including cross-sectional, case-control and cohort studies; (2) studies conducted in Ethiopia; (3) studies that reported prevalence and/or risk factors; (4) the outcome was mother-to-child HIV transmission; (4) both published and unpublished studies at any time; (5) studies used any HIV diagnosis approach. MTCT of HIV was defined as the proportion of the number of infants positive for HIV divided by the total HIV-exposed infants assessed. The prevalence in cohort studies the cumulative incidence was considered as prevalence, in which the number of new HIV infected cases divided by the overall sample size. Studies focused on the assessment of knowledge, attitude, and practice of MTCT without the outcome of interest of this study, program evaluation studies, studies with only abstracts, case studies, qualitative studies and citations without full-text were excluded.

### Study selection and quality assessment

All retrieved studies were exported to Endnote version 7 (Thomson Reuters, London) reference manager and duplicated studies were carefully removed. Two investigators (AE and AD) independently screened the titles and abstracts which were followed by a full-text review to determine the eligibility of each study. The disagreement was solved by consensus. Two independent reviewers (AE and SE) have assessed the quality of the studies. The quality of each article was evaluated using Joanna Briggs Institute (JBI) quality appraisal criteria adapted for studies reporting prevalence data, cross-sectional, cohort and case-control studies [37]. The following items were used to appraise cross-sectional studies: (1) inclusion criteria; (2) description of study subject and setting; (3) valid and reliable measurement of exposure; (4) objective and standard criteria used; (5) identification of confounder; (6) strategies to handle confounder; (7) outcome measurement; and (8) appropriate statistical analysis. The following items were used for appraising cohort studies: (1) similarity of groups; (2) similarity of exposure measurement; (3) validity and reliability of measurement; (4) identification of confounder; (5) strategies to deal with confounder; (6) appropriateness of groups/participants at the start of the study; validity and reliability of outcome measured; (8) sufficiency of follow up time; (9) completeness of follow-up or descriptions of reason to loss to follow-up; (10) strategies to address incomplete follow-up; and (11) appropriateness of statistical analysis. The following items were used for appraising case-control study: (1) comparable groups; (2) appropriateness of cases and controls; (3) criteria to identify cases and controls; (4) standard measurement of exposure; (5) similarity in measurement of exposure for cases and controls; (6) handling of confounder; (7) strategies to handle confounder; (8) standard assessment of outcome; (9) appropriateness of duration for exposure; and (10) appropriateness of statistical analysis. Studies considered low risk whenever fitted to 50% and or above quality assessment checklist criteria’s.

### Data extraction

Two independent reviewers (AE and TD) extracted the data and cross-checked to ensure consistency. Discrepancies were solved by discussion and repeating the procedure. Information about the author and year of publication, study area, study design, sample size, the prevalence rate of MTCT of HIV and AOR for identified risk factors were extracted. The reviewer contacted the corresponding author(s) for further information whenever pertinent data were missed from the included studies.

### Data analysis

A weighted inverse variance random-effects model [38] was used to estimate the overall pooled prevalence. The pooled AOR of rural residence, home delivery, mixed infant feeding, mothers ARV prophylaxis, PMTCT intervention, and infant ARV prophylaxis. Subgroup analyses according to the study geographical area and study design were employed to adjust the variations in the pooled estimate of the prevalence. The percentage of total variation across studies due to heterogeneity was assessed using an I^2^ statistic [39]. The values of I^2^ 25, 50, and 75% represented low, moderate and high heterogeneity respectively [39]. Publication bias across studies was checked using funnel plot and Egger’s regression test [40]. STATA version 14 (Stata Corp, College Station, TX, USA) statistical software was used for all statistical analysis.

## Result

### Characteristics of included studies

**Fig. 1:**
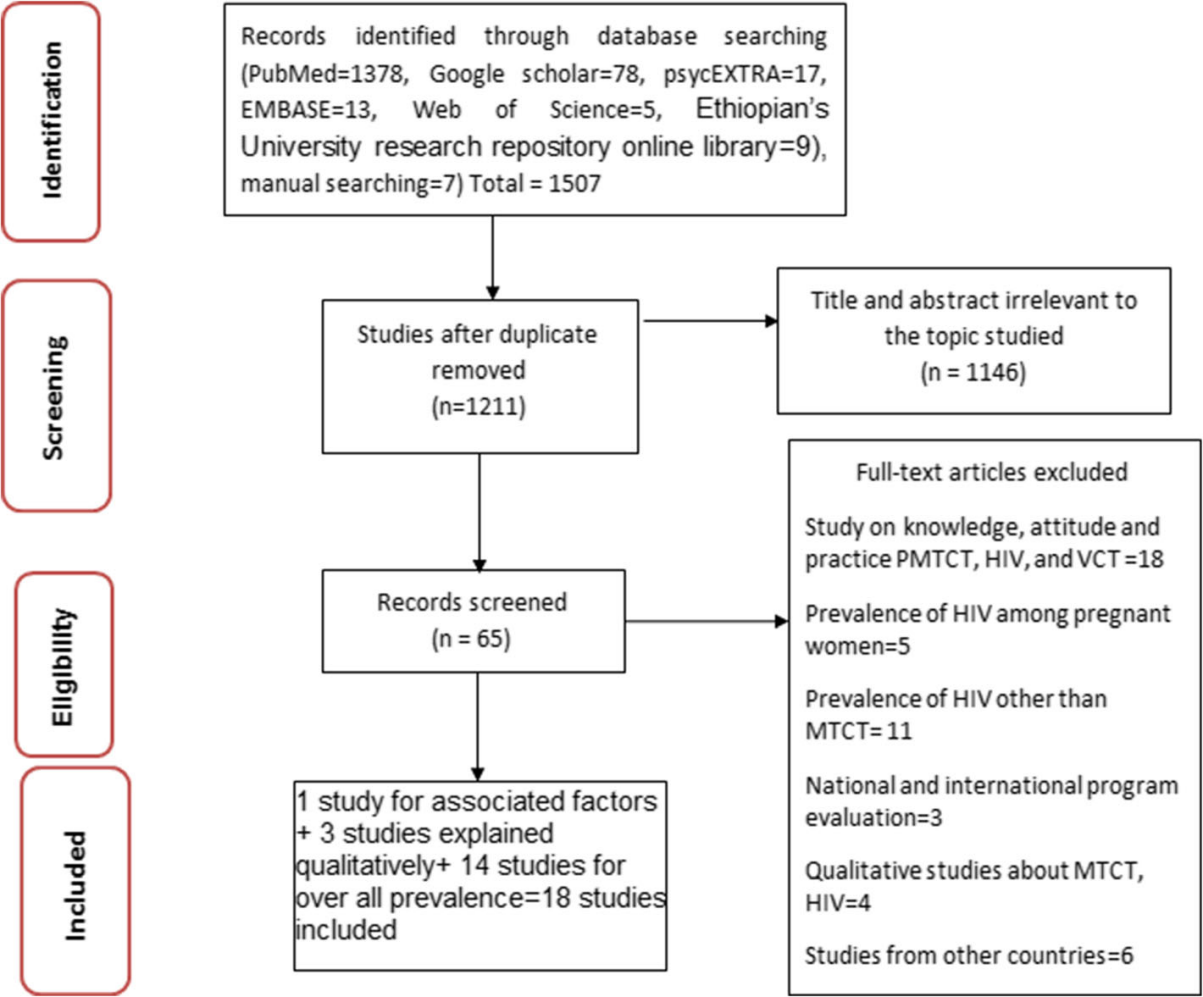
Studies selection process flow chart for the studies included in the analyses

The search strategy identified 1378 articles from PubMed, 78 articles from Google Scholar, 17 articles from psycEXTRA, 13 articles from EMBASE, 4 articles from Web of Science, 9 articles from Ethiopian’s University research repository online library, and 7 articles through manual search. First, 65 articles were selected for full-text review after duplicated (*n* = 296) and irrelevant studies based on the titles and abstracts (*n* = 1146) were removed. Then, the full-text review was performed and 47 articles were excluded for different reasons. Finally, 18 articles were found relevant to determine the prevalence and/or associated factors. Fig. 1 has shown the study selection process.

Among eighteen studies, one case-control [30], seven cross-sectional [18–20, 24, 27, 29, 35], and ten cohort studies [21–23, 25, 26, 28, 31–34] were found. Regarding the geographical area, five studies were conducted in Amhara region [20, 22–24, 27], five in Oromia [18, 21, 30, 32, 34], one in Southern Nation Nationalities and People (SNNPR) [26], six in Addis Ababa [19, 28, 29, 31, 33, 35] and one in Dire Dawa city [25]. Table 1 shows the characteristics of those studies.

**Table 1:** General characteristics of the included studies

Author/Year	Study Area	Study design	Sample size	Prevalence (95% CI)	Quality
Moges AN et al./2017 [23]	Amhara	Retrospective cohort	305	5.9(3.3–8.5)	Low risk
Tadele T et al./2014 [26]	SNNP	Retrospective follow up	457	4.2(2.3–6.0)	Low risk
Amare H et al./2014 [19]	Addis Ababa	Cross-sectional	159	32.1(24.8–39.4)	Low risk
Berhan Z et al./2014 [20]	Amhara	Cross-sectional	434	10.1(7.3–12.9)	Low risk
Birlie B et al./2016 [21]	Oromia	Retrospective follow up	146	17(10.9–23.1)	Low risk
Koye DN, Zeleke BM/2013 [22]	Amhara	Retrospective follow up	509	10(7.4–12.6)	Low risk
Wudineh F, Damtew B/ 2016 [25]	Dire Dawa	Retrospective cohort	382	15.7(12.1–19.3)	Low risk
Abdula M/2015 [26]	Oromia	Cross-sectional	130	7.7(3.1–12.3)	Low risk
Asmamaw Y et al./2017 [27]	Amhara	Cross-sectional	313	3.8(1.7–5.9)	Low risk
Tigabu Z, Wasie B/2016 [24]	Amhara	Cross-sectional	484	12.4(1.5–9.5)	Low risk
Girma M/2016 [28]	Addis Ababa	Prospective cohort	435	0.7(−0.08–1.5)	Low risk
Shargie MB et al./2011 [29]	Addis Ababa	Cross-sectional	118	6.8(2.3–11.3)	Low risk
Burusie A, Deyessa N/2015 [30]	Oromia	Case-control	424	Not applicable	Low risk
Mirkuzie AH et al./2010 [31]	Addis Abba	Retrospective cohort	896	11.8 (9,7–13.9)	Low risk
Derebe G et al./2014 [32]	Oromia	Retrospective cohort	426	9.6 (6.8–12.4)	Low risk
Mirkuzie AH et al./2011 [33]	Addis Ababa	Prospective cohort	71	8.4 (1.9–14.8)	Low risk
Kumela K et al./2015 [34]	Oromia	Retrospective cohort	180	15.5 (10.2–20.8)	Low risk
Negash TG, Ehlers VJ/2016 [35]	Addis Ababa	Cross-sectional	384	6.0 (3.6–11.3)	Low risk

### Quality of the included studies

One study was assessed using JBI checklist for case-control studies [30], ten studies [21–23, 25, 26, 28, 31–34] using JBI checklist for cohort studies, and seven studies [18–20, 24, 27, 29, 35] using the JBI checklist for cross-sectional studies. None of the studies were excluded based on the quality assessment criteria (Table 1).

### Meta-analysis

#### Publication bias

**Fig. 2:**
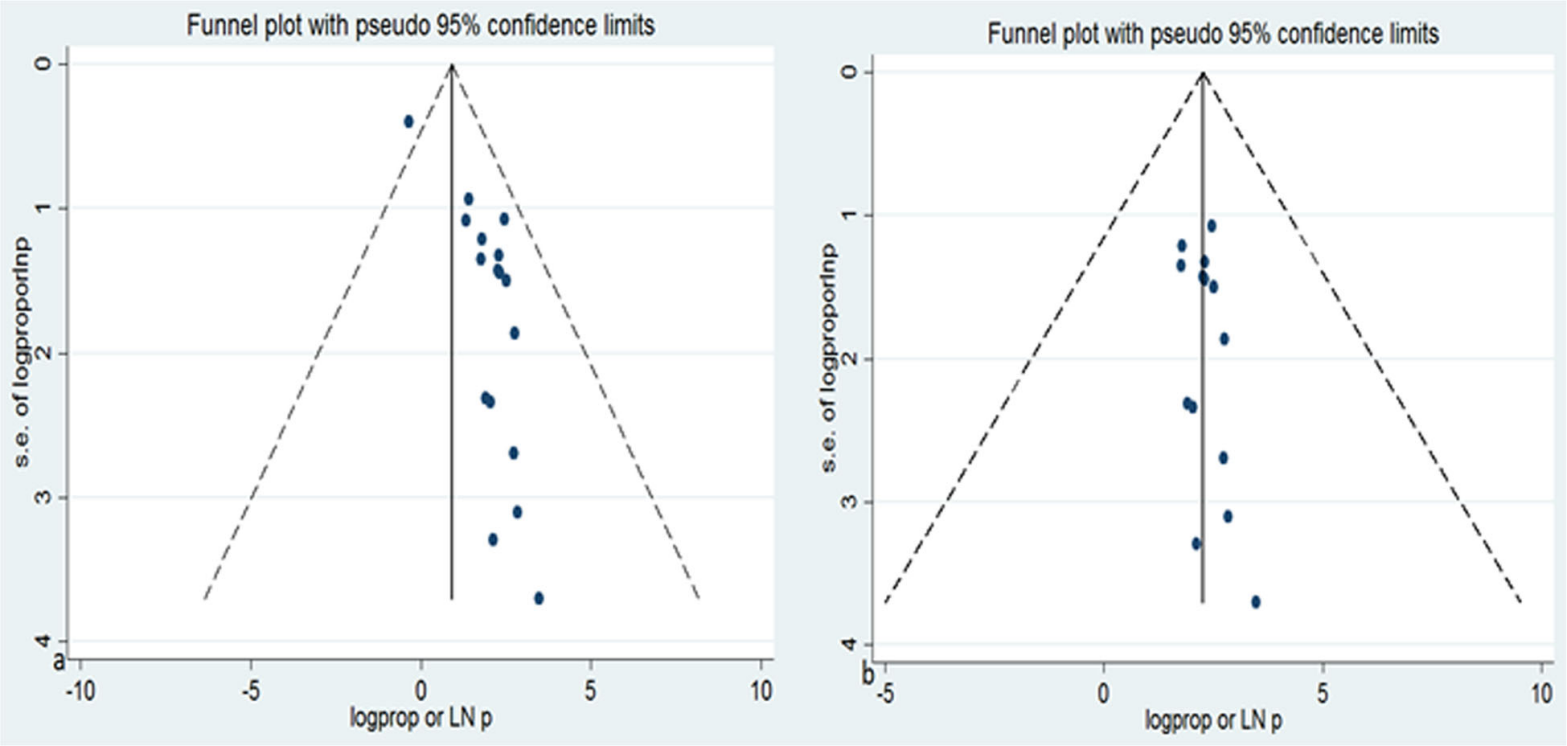
Funnel plot before adjustment (**a**) and after adjustment (**b**) for publication bias, Logprop or LNP (log of proportion) represented in the x-axis and standard error of log proportion in the y-axis

Three studies [26–28] were excluded from prevalence estimation after checking funnel plot and the significance of Egger’s regression test. However, they were not excluded from meta-analysis for risk factors. Significant publication bias with an Egger’s regression *p*-value< 0.001 was seen when all studies considered (Fig. 2a). After adjustment, Egger’s regression p-value was 0.206, indicated a reduced publication bias (Fig. 2b).

#### Qualitative description

Primarily, among eighteen studies, the one was case-control study was not considered in the prevalence estimation. The other three studies [26–28] were excluded from the pooled analysis step-by-step until p-value in the Egger’s regression test scored greater than 0.05. These studies were: Girma M [28] report the prevalence of MTCT of HIV was 0.7% in Addis Ababa whereas Asmamaw et al. [27] reported 3.8% in Amhara region. In addition, Tadele T et al. [26] found 4.16% prevalence of MTCT of HIV in SNNPR region.

#### Prevalence of MTCT of HIV

**Fig. 3:**
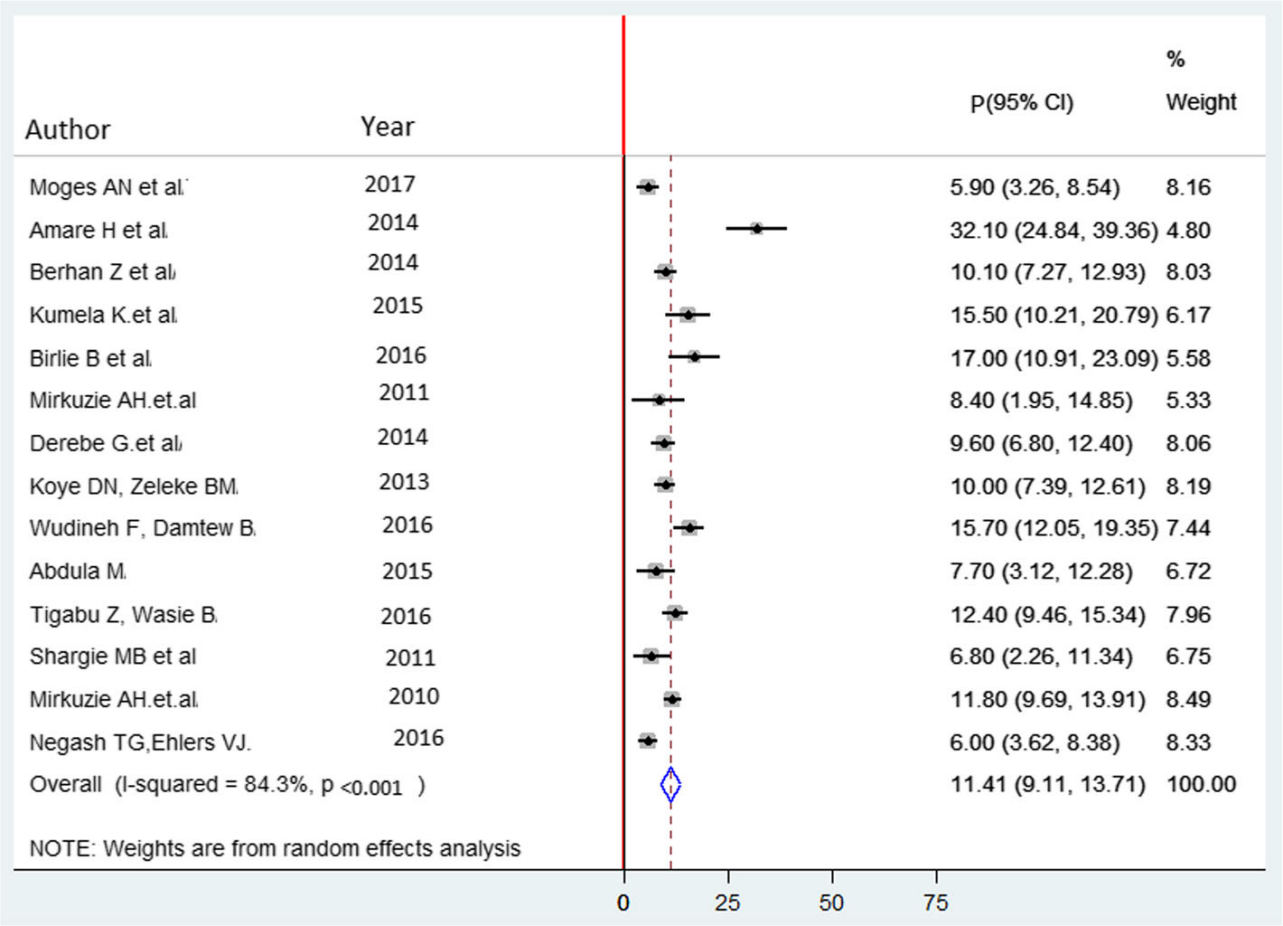
Forest plot of the prevalence with corresponding 95% CIs of the fourteen studies on MTCT of HIV. The midpoint and the length of each segment indicated prevalence and a 95% CI whereas the diamond shape showed the combined prevalence of all studies

Consequently, fourteen studies [18–25, 29, 31–35] were included in the final meta-analysis. The prevalence of MTCT of HIV ranges from 5.9% in Amhara region [23] to 32.1% in Addis Ababa [19]. The pooled prevalence was 11.4% (95% Confidence Interval) CI = 9.1 to 13.7, I^2^ = 8.43%; *p* < 0.001) (Fig. 3). Egger’s regression test *p*-value = 0.206.

**Fig. 4:**
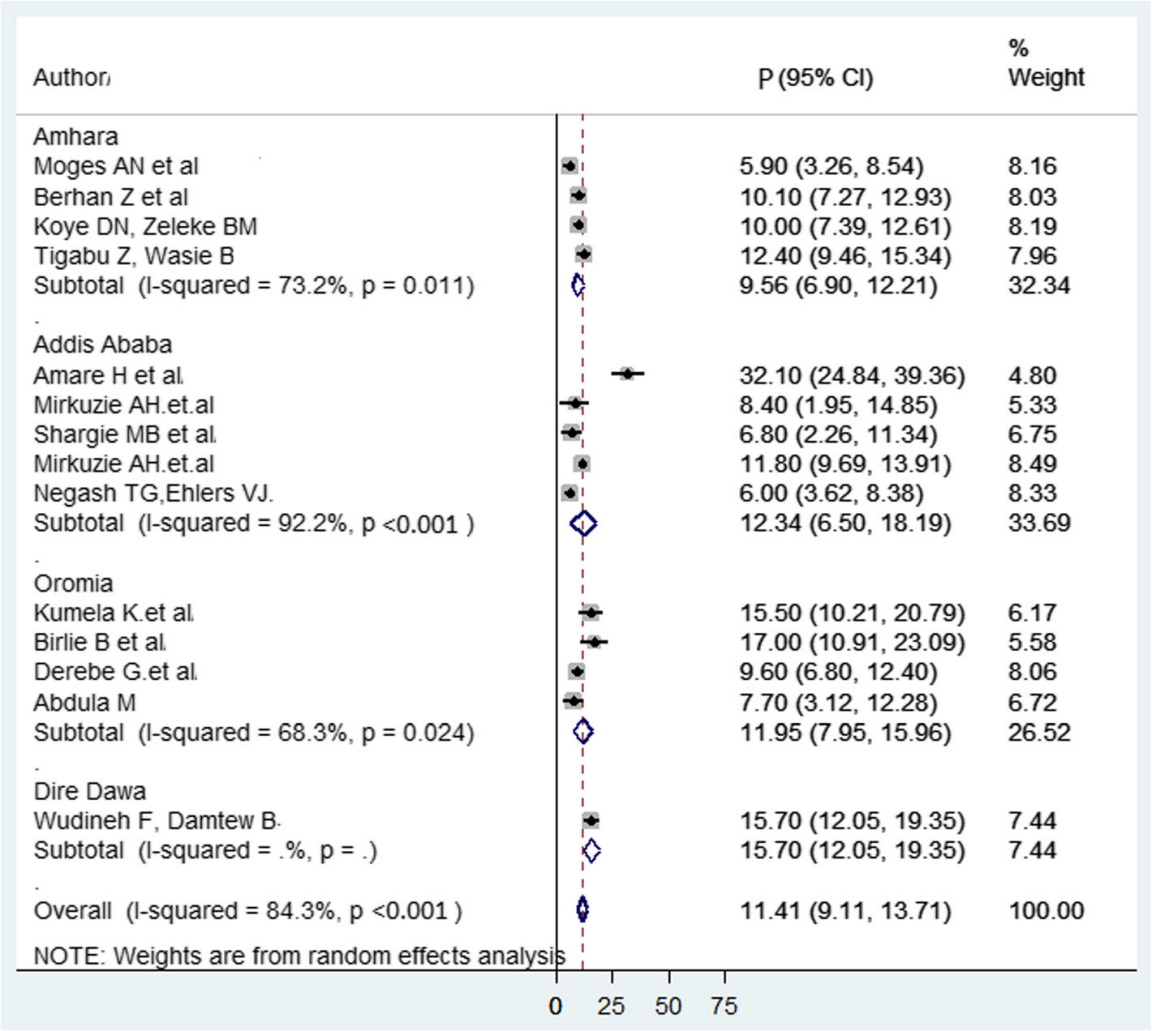
Forest plot of the prevalence with corresponding 95% CIs of the subgroup analysis based on the regions, where the studies done. The midpoint and the length of each segment indicated prevalence and a 95% CI whereas the diamond shape showed the combined prevalence

We performed subgroup analysis by region and study design. Consequently, MTCT of HIV prevalence was 15.7% in Dire Dawa, 12.34% in Addis Ababa, 11.95% in Oromia, and 9.56% in Amhara region (Fig. 4).

**Fig. 5:**
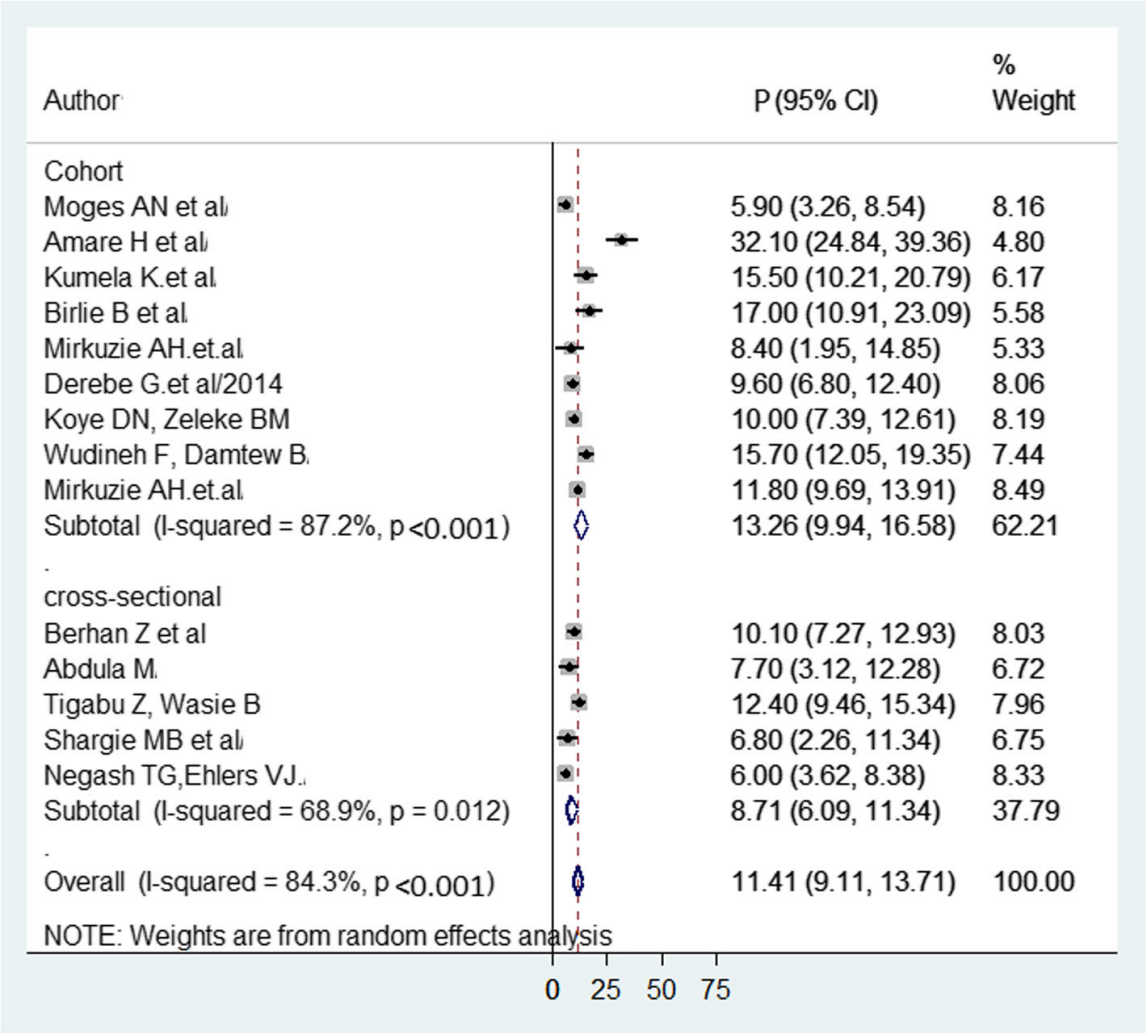
Forest plot of the prevalence with corresponding 95% CIs of the study design. The midpoint and the length of each segment indicated prevalence and a 95% CI whereas the diamond shape showed the combined prevalence

In addition, the higher prevalence was (13.3%) reported among cohort studies. The detailed results are illustrated in Fig. 5.

#### Risk factors for MTCT of HIV

Based on the review done associated factors were categorized into three thematic areas. These were: (1) socio-demographic, (2) prenatal, intranatal and postnatal, and (3) clinical and drugs-related factors.

#### Socio-demographic related factors

**Fig. 6:**
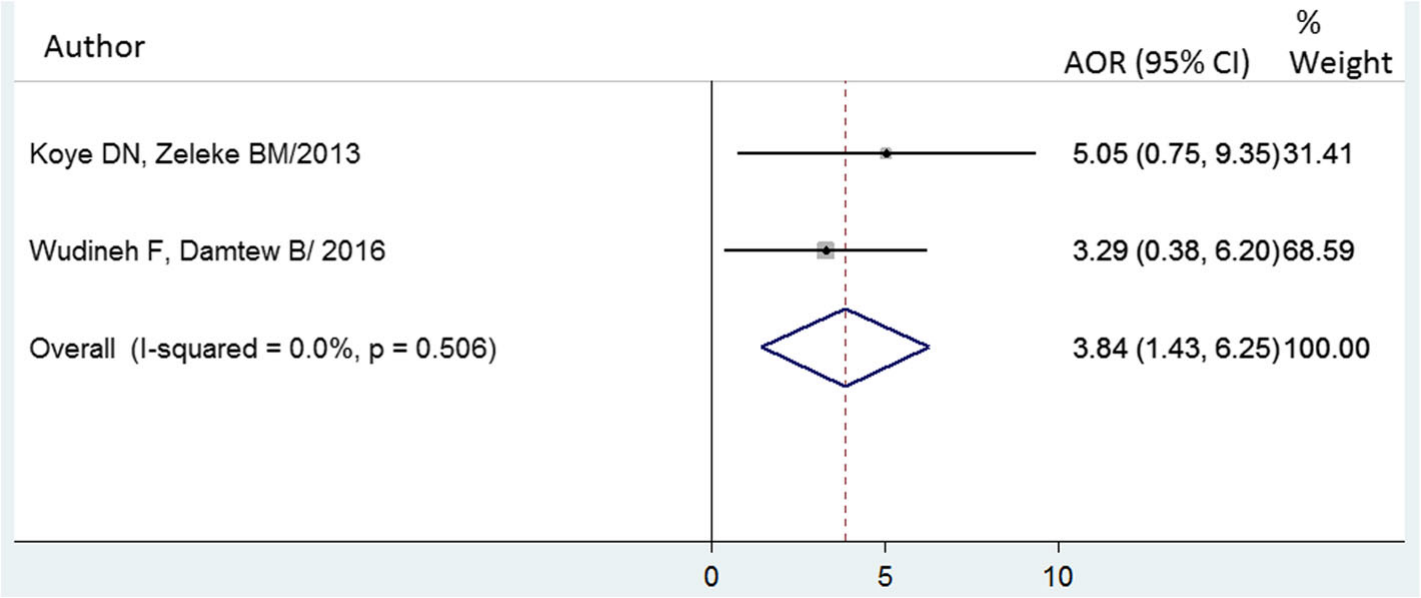
Forest plot of the adjusted odds ratios with corresponding 95% CIs of studies on the association of rural residence and MTCT of HIV. The midpoint and the length of each segment indicated an AOR and a 95% CI; the arrow showed the widest CI; and the diamond shape showed the combined AOR of all studies

Those children who were born from mothers age greater than 27 years were more likely (AOR = 5.4, 95% CI = 1.15, 25.70) to acquire HIV infection as compared to those with less than 27 years [23]. Two studies [22, 25] reported being rural residence was associated factor of MTCT of HIV. The pooled AOR of MTCT of HIV in infant from rural versus urban residence was 3.8 (95% CI = 1.4 to 6.3, I^2^ = 0.0%, *p* = 0.506) (Fig. 6).

#### Prenatal, Intranatal, and post natal-related factors

Mothers who became pregnant after they knew their HIV positivity (AOR = 0.22, 95%CI = 0.049, 096) were less likely to transmit HIV to their infants [23]. Additionally, the rate of HIV infection was higher among those mothers who knew their HIV sero-positivity during pregnancy (AOR = 4.71, 95% CI = 1.39–15.93) and after delivery (AOR = 4.46, 95%CI: 1.40–16.22) as compared with those who knew before getting pregnant [30]. The presence of mothers’ illness during pregnancy (AOR = 20.4, 95%CI: 3.1–25.7) [26] and absence of maternal antenatal care visit (AOR = 4.6, 95%CI: 1.17–17.99) [27] were also contributing factors to MTCT of HIV.

HIV exposed infants who were enrolled in the follow-up clinic lately (AOR = 2.89, 95% CI: 1.35, 6.21) were more likely to have HIV infection [22]. A mother who had cracked nipple and/or mastitis while lactating (AOR = 13.05, 95%CI: 1.23–138.21) was also found a significant predictor [30].

**Fig. 7:**
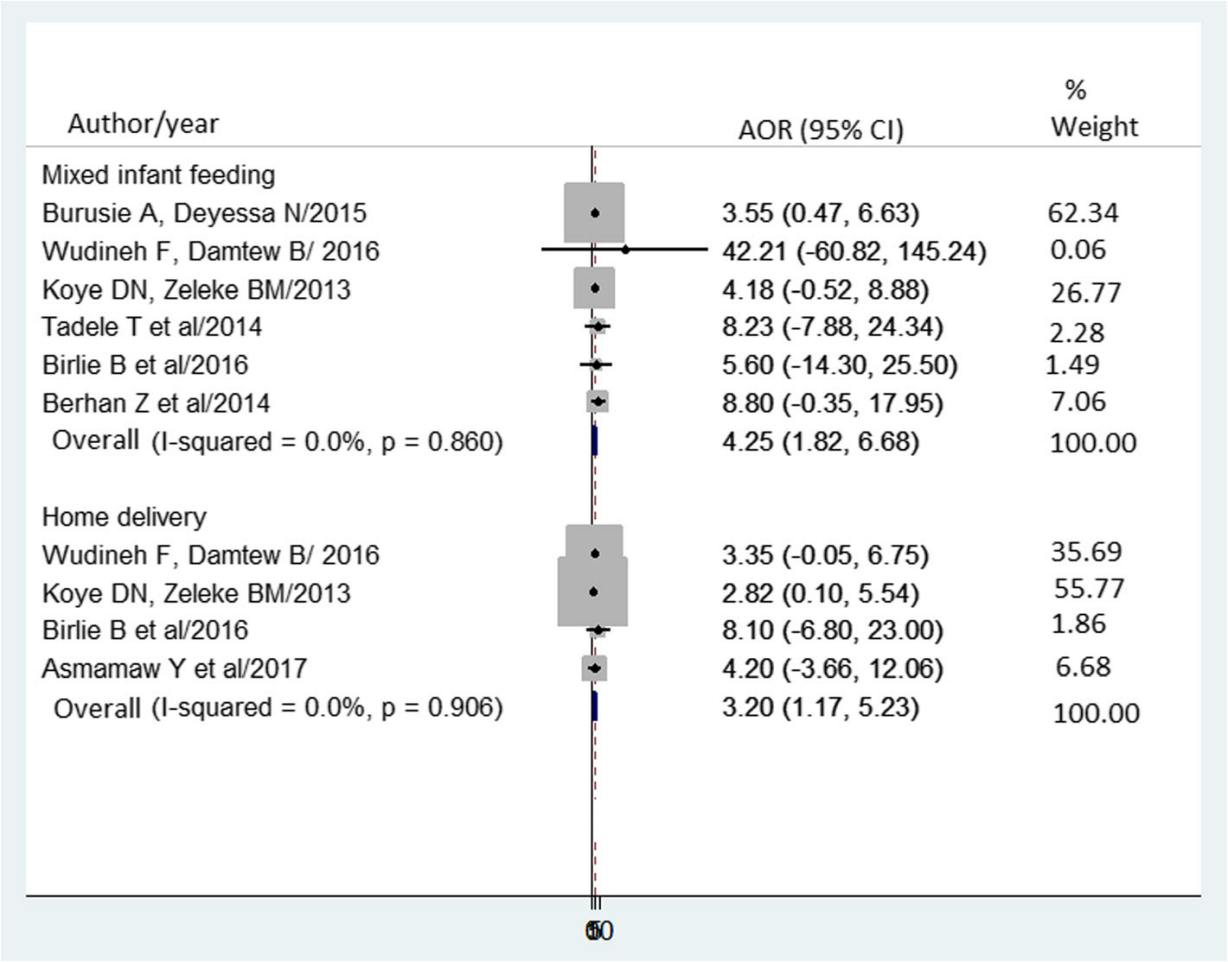
Forest plot of the adjusted odds ratios with corresponding 95% CIs of studies on the association of home delivery and mixed infant feeding with MTCT of HIV. The midpoint and the length of each segment indicated an AOR and a 95% CI; the arrow showed widest CI; and the diamond shape showed the combined AOR of all studies for each variable

Four studies [21, 22, 24, 25] showed a significant association between place of birth and MTCT of HIV. The pooled AOR of MTCT of HIV for infants born at home versus health institution was 3.2 (95% CI = 1.2 to 5.2, I^2^ = 0.0%, *p* = 0.906) (Fig. 7). Egger’s regression test was showed a *p*-value of 0.055.

Six studies [20–22, 25, 26, 30] showed a significant association between infant feeding practice and MTCT of HIV. The pooled AOR of MTCT of HIV in mixed feeding versus exclusive breastfeeding practice was 4.3 (95% CI = 1.8 to 6.7, I^2^ = 0.0%, *p* = 0.860) (Fig. 7). Egger’s regression test was showed a *p*-value of 0.085.

#### Clinical and drugs-related factors

Delayed HIV diagnosis (AOR = 2.7, 95% CI = 1.3, 29.4) [20], mothers being on late AIDS stage (AOR = 5.8; 95% CI: 1.6–16.5) [21], mothers with CD4 cell count < 200 (AOR = 7.65, 95%CI: 3.20–18.31), and 201–500 (AOR = 4. 07, 95%CI: 1.90–8.71) cells/μl during lactation [30] were found more likely to transmit HIV to their child.

**Fig. 8:**
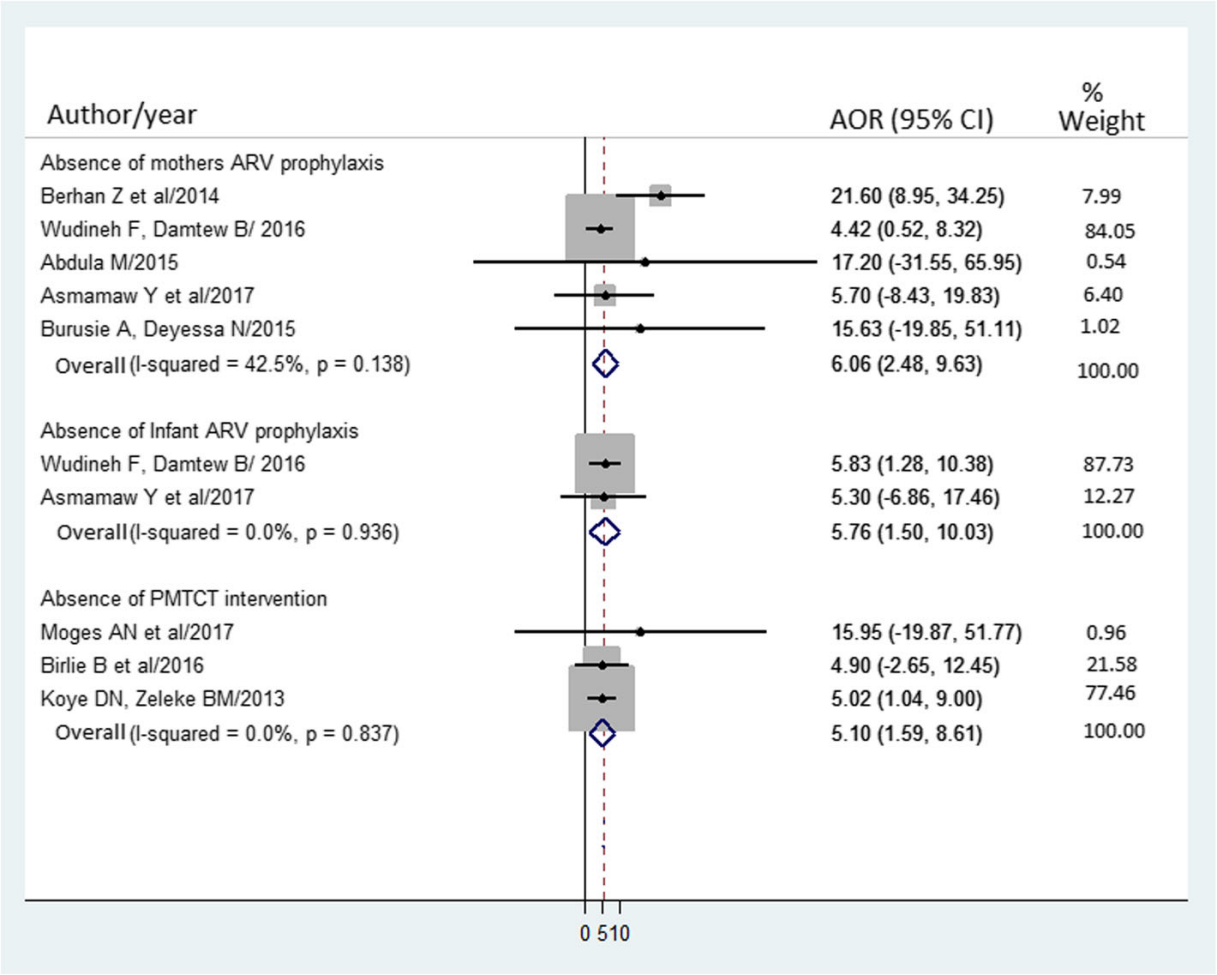
Forest plot of the adjusted odds ratios with corresponding 95% CIs of studies on the association of absence of mother ARV prophylaxis, absence of infant ARV prophylaxis, and absence of PMTCT intervention with MTCT of HIV. The midpoint and the length of each segment indicated an AOR and a 95% CI; the arrow showed widest CI; and the diamond shape showed the combined AOR of all studies for each variable

Three studies [21–23] showed a significant association between PMTCT utilization and MTCT of HIV. The pooled AOR of MTCT of HIV in infants whose mother couldn’t get PMTCT intervention was 5.1 (95% CI = 1.6 to 8.6, I2 = 0.0%, *p* = 0.837) (Fig. 8). Egger’s regression test was showed a *p*-value of 0.343.

Five studies [20, 25–27, 30] showed a significant association between mothers on ART prophylaxis during pregnancy and/or breastfeeding and MTCT of HIV. The pooled AOR of MTCT of HIV in infants whose mothers didn’t receive prophylaxis during pregnancy and/or breastfeeding was 6.1 (95% CI = 2.3 to 9.6, I2 = 42.5%, *p* = 0.138) (Fig. 8). Egger’s regression test was showed a p-value of 0.204.

Two studies [25, 27] showed a significant association between infants ART prophylaxis and MTCT of HIV. The pooled AOR of MTCT of HIV in infants didn’t receive ARV prophylaxis was 5.8 (95% CI = 1.5 to 10.0, I2 = 0.0%, *p* = 0.936) (Fig. 8).

## Discussion

Our meta-analysis aimed to estimate the pooled prevalence of MTCT of HIV and its associated factors in Ethiopia. In this meta-analysis, the overall pooled prevalence rate of MTCT of HIV was 11.4%. In addition, sociodemographic, natal, and clinical and drug-related factors were found to be the predictors of MTCT of HIV.

The prevalence of MTCT of HIV in the current study was higher than 2013 United Nations Program on HIV/AIDS reports in South Africa (6%) and in Botswana (2%) [41]. Low maternal adherence to antenatal care utilization, extensive home delivery, less availability and accessibility of PMTCT interventions and HIV counseling in remote areas, inconsistent availability of infrastructures like roads, light and water and prevailing pre-lacteal feeding habit might cause the higher rate of MTCT of HIV in Ethiopia. Low level of knowledge and awareness of mothers about MTCT of HIV might also attribute to high HIV infection rate among infants in Ethiopia [42, 43]. Moreover, MTCT of HIV has been eliminated in some countries, like Cuba, Belarus, Armenia, and Thailand [44]. This might be due to, in these countries, minimized of the pregnant women practiced unprotected sex, women with HIV in those countries didn’t breastfeed their babies, availability of best suited safe and healthy alternative baby formula, good attitudes and perceptions to use HIV drugs during pregnancy, high level of early HIV test before getting pregnant and/or during pregnancy, and persistence implementation of PMTCT after the infants delivered safely.

Although the current finding showed the high burden, it is lower than 2013 UNAIDS reports in Burkina Faso (22%) and in Ethiopia (25%) [41]. The possible reasons for such discrepancy might be related to year of the study, and an emerging of new strategies and improvement on HIV prevention and control activities.

The subgroup analysis revealed that there was a significant variation among regions. Infants born from HIV-positive mothers in Amhara region had lower rates of MTCT of HIV compared to Addis Ababa and Oromia regions. However, this finding was inconsistent with the 2016 Ethiopia Demographic Health Survey (EDHS) reports in Addis Ababa and Amhara region [16]. This discrepancy might be due to the fact that there might be the change in the epidemiological transitions of diseases, on and off interventions as per the prevailing HIV cases, the difference in HIV-test coverage, and the difference of awareness to HIV.

According to this study, infants from the rural residence were nearly four times more likely to acquire HIV infection from their mothers. This could be due to living in rural area of Ethiopia, low knowledge to MTCT of HIV, the high proportion of mothers unaware of their HIV status, lack of clinic-based education and counseling [45], lower level of education, belonging to lower wealth, and not exposed to mass media [46].

In this study, infants delivered at home were nearly three times more likely to get HIV infection compared to infants delivered at health institutions. This finding was in agreement with a study conducted in Nigeria [47] and Zimbabwe [48]. This could be due to home delivery lack implementation of HIV prevention strategies as it does in the health institution. The 90% of HIV-infection among infants born from seropositive mothers is higher during labor and delivery [5], particularly in the absence of integrated HIV services. The previous study [49] in Africa showed infants who delivered at home were more prone to many harmful traditional practices that promote HIV-infection rate, such as cord-cutting by shared razor, placental blood contamination, uvulectomy, unplanned circumcision, pre-lacteal feeding, and breastfeeding from unexamined nipples.

This study also showed that HIV-exposed infants who didn’t take ARV prophylaxis and whose mothers didn’t receive prophylaxis during pregnancy and/or breastfeeding were nearly six times more likely to get HIV-infection. This finding was in line with a study conducted in Cote'devore [50], South Africa, and sub-Saharan Africa [51, 52]. There is also evidence that showed not initiating ARV prophylaxis to the infant is a risk factor for MTCT of HIV [53, 54]. This is due to the fact that without ARV drugs a potential effect of HIV transcription, replication, and fusion increased in the human body [55]. Besides, those infants whose mother couldn’t get PMTCT intervention were 5 times more likely to have HIV infection. This finding was in agreement with studies done in Kenya [56, 57]. PMTCT strategies are considering prevention of HIV infection among women, prevention of unwanted pregnancy, antenatal protection of fetus, test and counseling of pregnant women, ARV prophylaxis, and treatment of pregnant women. Therefore, MTCT of HIV could highly observable among those mothers lacked PMTCT interventions.

Mixed infant feeding practice also identified as a key predictor of high rate of MTCT of HIV; infants who received mixed feeding were seven times more likely to acquire HIV infection compared to those exclusively breastfed. This finding agreed with studies in South Africa [58] and Zimbabwe [59]. Mixed feeding practices may cause laceration of gastrointestinal mucosa which would create a favorable entry of the virus into the bloodstream.

MTCT continues to be a devastating clinical and/or public health burden in Ethiopia. Adequate emphasis has not been given on this pandemic which might lead to increased hospitalizations, cost of healthcare services and reduction of the overall economic structure of the nation. It could be reduced if all women delivered at health institutions. To achieve the WHO’s end AIDS strategy [11], Ethiopia planned to create “HIV-free generation by the year 2020” [13] and implementing the health policy that focused on PMTCT and other infectious diseases. However, the burden of MTCT of HIV remains high in the Ethiopian population. Thus, the finding of this study would be important to develop further HIV control interventions and may have a significant impact on health service resource utilization. It also contributes to the growing need for undertaking ART. It will have direct or indirect importance in providing information to the Joint United Nations Programme on HIV/AIDS and partners, 90–90-90 targets; 90% of all HIV-positive persons identified, provide antiretroviral therapy (ART) for 90% of those diagnosed, and achieve viral suppression for 90% of those treated by 2020.

### Strength and limitation

This study as it is the first systematic review and meta-analysis that provided the national prevalence estimate on MTCT of HIV. In addition, the effects of three key predictors of MTCT of HIV were estimated. On the other hand, given the limited number of studies, the result may not represent the national figure. Furthermore, the time-trend analysis was not conducted because studies were not available in all the year.

## Conclusion

The MTCT rate of HIV was high. Urban residence, home delivery, not taking antiretroviral prophylaxis, the absence of PMTCT intervention, and mixed infant feeding practices were significant predictors of MTCT in Ethiopia.

## Additional files

Additional file 1: Research check list. (DOC 63 kb)

Additional file 2: Search strategy. (DOCX 16 kb)

## Abbreviations

AIDS: Acquired Immunodeficiency Syndrome
ARV: Antiretroviral
CI: Confidence Interval
HIV: Human Immunodeficiency Virus
MTCT: Mother-to-Child Transmission
OR: Odds Ratio
PMTCT: Prevention of Mother to Child Transmission
