# Supplementary material for: A systematic review and meta-analysis of vertical transmission route of HIV in Ethiopia
Source: BMC Infect Dis. 2018 Jun 22;18:283. doi: 10.1186/s12879-018-3189-3 (PMC6013937; doi:10.1186/s12879-018-3189-3)
Supplement: Supplementary file 2 — Search strategy. (DOCX 16 kb) [file 12879_2018_3189_MOESM2_ESM.docx]

Additional file 2: PubMed search strategy

| Search terms and /or phrases | Items found |
| --- | --- |
| 1. (HIV OR human immunodeficiency virus[MeSH Terms] OR AIDS[MeSH Terms] OR Acquired immunodeficiency syndrome[MeSH Terms]) AND (vertical transmission OR MTCT[MeSH Terms] OR mother to child OR pmtct OR prevention) AND (factors OR determinants[MeSH Terms] OR predictors[MeSH Terms] OR enablers[MeSH Terms] OR barriers[MeSH Terms]) AND (ethiopia) | 233 |
| 1. [("HIV"[All Fields] OR "acquired immunodeficiency syndrome" [MeSH Terms]) AND ("infectious disease transmission" [All Fields]) AND (“vertical" [All Fields]) OR ("infectious"[All Fields] AND "disease"[All Fields] AND "transmission"[All Fields] AND "vertical"[All Fields]) OR "vertical infectious disease transmission"[All Fields] OR ("vertical"[All Fields] AND "transmission"[All Fields]) OR "vertical transmission"[All Fields]) OR (("mothers"[MeSH Terms] OR "mothers"[All Fields] OR "mother"[All Fields]) AND ("child"[MeSH Terms] OR "child"[All Fields])) OR PMTCT[All Fields] OR prvention[All Fields]) AND factors[All Fields] AND ("ethiopia"[MeSH Terms] OR "ethiopia"[All Fields])] | 378 |
| 1. [(HIV OR human immunodeficiency virus [MeSH Terms] OR AIDS [MeSH Terms] OR Acquired immunodeficiency syndrome [MeSH Terms]) AND (factors OR determinants [MeSH Terms] OR predictors [MeSH Terms] OR enablers [MeSH Terms] OR barriers [MeSH Terms]) AND (Ethiopia)] | 767 |
